# A Case of Albuminuric Eclampsia*Read before the Medical Society of the county of Albany, New York, April 28th, 1875

**Published:** 1875-07

**Authors:** G. L. Ullman

**Affiliations:** Albany, N. Y.


### A CASE OF ALBUMINURIC ECLAMPSIA.*

BY G. L. ULLMAN, M.D., ALBANY, N. Y.

W. C. P. Mall, æt. 25, American, when about ten years of age, had, as was supposed, two epileptic convulsions. Since that time had never enjoyed robust health, though he had been quite constant at his business. He applied to me on the evening of January 17th, complaining of a brow headache, which at times would be torturing; feeling slightly depressed with languor and a disinclination to exertion ; appetite fair, sleep a little disturbed at night, and the bowels in a torpid condition ; a moderately placid expression of the countenance, with pale and sallow skin ; frequently would be annoyed with muscæ volitantes, which, I learned, was supposed to have been occasioned by exposing the eyes to a brilliant flame from a gas jet, which was used directly in front of him for the purpose of performing his duty—working steadily in this position for hours. There was exophthalmus, which, however, had existed for some time ; no external congestion ; occasional obscured sight or a cloud covering the field of vision. I directed him to use caution in pro-

*Read before the Medical Society of the county of Albany, New York, April 28th, 1875.

tecting his eyes with a shade while at work evenings, and ordered compound tincture of gentian, spirits of nitric ether and dilute phosphoric acid.

The next morning, January 18th, I was sent for, and found my patient lying on a sofa, apparently suffering from an intense headache, morbid sensibility to sight, had vomited, pulse a little over normal and breath very offensive. Prescribed a dose of compound cathartic pills and a mixture containing potass. bromide—gr. x. to a dose, and spt. ether nit., gtts. xv.—to be taken every three hours.

Late in the afternoon the attention of his mother was called to a slight noise made by him. He was found with his head slightly turned to the right; his arms had at first tonic spasms, and afterwards clonic spasms. He was removed from the sofa and placed in bed, remaining unconscious for several hours. Pulse was rapid and irregular, breathing heavy, difficult and stertorous. A mustard paste was applied to the nape of the neck and along the spine; bottles containing hot water to the feet; a cloth wrung in cold water and placed on the head, and an enemata administered.

After a time he could be easily aroused by mentioning his name sharply, and would ask for a drink of water. Instead of water being given him during the evening, a bottle of citrate of magnesia was procured, and he was allowed to take that as a refrigerant, and more particularly to induce a cathartic effect—being desirous to obtain an evacuation from the bowels, for the pills given in the morning as yet had no effect, nor the enemata sufficiently. Later, however, an evacuation was produced and some relief was experienced. The night was passed restlessly and the snoring still continuing.

The next day, January 19th, remained pretty much in a quiet state, and apparently sleeping considerable, though drinking quite frequently. Pulse quick, respiration irregular, and passing considerable urine, which produced much froth in the vessel, which led me to suspect albumen. On examination, it was found to be highly albuminous. I inquired of his mother as to how long this frothy condition had been previously noticed, and she informed me it had been so for some time past, and would retain this frothy appearance for several hours—in fact, for days—she attributing it to that of drinking beer.

January 23d. Condition much improved. He would converse, though stupid at times. The mixture of bromide potass. and nitre was continued, and cit. of ferri and quinine also was given.

After a few days recovery was so complete as to permit him to resume his daily vocation, however still looking pale, sallow and wan. From time to time his urine was examined, and found to contain albumen more in quantity sometimes than at others. He was placed on tinct. ferri ch'or. in ten-drop doses, three times daily, and followed later by alternating the diuretic mixture, containing digitates, squills, nitre and buchu, and seemed to improve a little; frequently visiting me at my office and bringing a bottle of urine for examination, saying that he felt good and that his appetite was excellent. He afterwards told me, however, that he remembered nothing that occurred during the thirty-six days that elapsed before I was again consulted concerning the case, although he performed unusual business duties, and, as I have said, visited me on several occasions.

The day preceding the second convulsion he felt a peculiar sensation, originating in the head and traversing the spine, but complained of no unpleasantness resulting.

The next day, February 23d, there was great congestion of the conjunctiva, superabundaut secretion ot tears, with intolerance to light, edœma of the lid and pain in the frontal prominence. Tea leaves were being applied to the eyes, and I substituted a cold rinse of sulph. zinc, with cold compresses, and had repeated the bromide potass and nitre, with the addition of the iodide internallv. In the afternoon, about 6:30, was seized with another convulsion, he being affected in a similar manner as the previous one; intermittent pulse beating 104; respiration imperfect, with stertorous breathing. Renewed the mu [illegible]rd to the nape of the neck and spine, with foot bath and cold [illegible]fusions to the head.

24th. No apparent change excepting oscillations of the eye-ball.

25th. Pulse remaining high; temperature 101 in the evening; urine scanty in secretion and highly albuminous, coagulating under the application of heat and nitric acid; tongue brown and dry; evacuations from bowels natural and breath very offensive; red patches of echymosis spread over the sclera.

26th. At 5 P. M. temperature 99; pulse considerably lowered, at a normal beat; urine again abundant; eyes appeared suffused with tears, but, upon examination, showed the fluid was beneath and not upon the mucous membrane; the upper lip was swollen and some puffiness about the eyes.

27th. Pulse 68; temperature normal; skin moist, accompanied with a peculiar odor arising therefrom.

28th. Pulse 68; a mucous diarrhœa; tongue still bearing a brown coat, and some irregularity in respiration and circulation.

March 1st. Mind clearer, with some signs of returning to consciousness; pulse 69; no diarrhœa, having been checked with sub-nitrate of bismuth; much thirst; cream tarter water was allowed to be drank freely; urine passed in large quantities, straw-colored and still retaining the frothy appearance.

2d. A little improvement, and could be easily aroused.

3d. Pulse 70. The cloud which obscured his sight was broken and he was able to recognize friends. He says his head is not right; head aches and is embarassed in speech; eyes improved; echymosis disappearing; specific gravity of urine, 1005; acid reaction; microscope only showed urates and large epithelial scales.

From this date my patient steadily improved, and in about a fortnight was sufficiently able to again get out, though more feeble from this attack than the previous one. In the meantime the urine was examined and found still to contain albumen.

Within a few days, however, he was again taken ill, and obliged to take to bed. From this on he gradually grew worse. After a time ascites developed itself, soon followed by hydropericardum. A cough was present, and later a shortness of breath; dyspnoea became difficult; a cough mixture, in addition with Hoffman's anodyne, was given with some benefit. Later the dyspnoea became so difficult that it was only relieved—and thereby prolonged his life—with small doses of hydrate of chloral, which [illegible]ted splendidly for the time being. The symptoms gradually grew worse, and after a short time he was relieved of his suffering by death.

Upon an examination of the family history of this patient, I ascertained that the father, grandfather and two uncles had died victims of Bright's disease.

